# Direct Immunofluorescence in Oral Lichen Planus and Related Lesions: Sensitivity, Specificity, and Diagnostic Accuracy in a Single Diagnostic Center in Poland

**DOI:** 10.3390/dj12120396

**Published:** 2024-12-06

**Authors:** Katarzyna Osipowicz, Konrad Szymański, Ewelina Pietrzyk, Emilia Milczarek, Cezary Kowalewski, Renata Górska, Katarzyna Woźniak

**Affiliations:** 1 Klinika OT.CO (OT.CO Clinic), 00-716 Warsaw, Poland; 2 Department of Dermatology, National Medical Institute of the Ministry of the Interior and Administration, 02-507 Warsaw, Poland; 3 Department of Mucosal and Periodontal Diseases, University Stomatology Center of the Medical University of Warsaw, 02-097 Warsaw, Poland

**Keywords:** oral erosions, oral lichenoid lesions, histopathology, DIF

## Abstract

**Objectives:** Our study aimed to establish the basic reliability parameters of direct immunofluorescence test results in patients with oral lichen planus. **Methods:** We conducted an evaluation of individual antibody classes in the DIF and ELISA (BP180 antigen), comparing these results with the classical histopathological (HP) examination in a group of patients treated within the standard healthcare in our clinic. **Results:** Among 66 participants with oral changes indicative of LP, only 50% received histopathological confirmation of the LP diagnosis. Among those with a DIF profile entirely typical for LP (C3+, F+), 57.1% had a positive HP result. Fibrinogen deposits were identified in 42.4% and 36.4% of individuals with positive HP results for F1 and F2, respectively; 78.8% of patients with negative HP and 57.6% with positive HP exhibited no fibrinogen deposits. Simultaneous positivity for F1 and F2 occurred in all cases where F1 was positive. HP confirmed positive DIF for C3 in 50% of cases. Fibrinogen deposits demonstrated the highest diagnostic accuracy (61%). Sensitivity and specificity for fibrinogen deposits were 36% and 42% for F1 and 79% and 82% for F2. The positive predictive values were 67% for F1 and 67% for F2, while the negative predictive values were 58% for F1 and 56% for F2. Overall diagnostic accuracy was reported at 61% for F1 and 59% for F2. **Conclusions:** Our data indicate the complementarity of HP and immunological test results and the necessity of using both methods together in cases of doubt.

## 1. Introduction

Oral lichen planus (OLP) is a chronic inflammatory and autoimmune disorder that affects the oral cavity, with a prevalence of approximately 1% in the population [1]. Usually, the diagnosis of OLP is based on clinical examination supported by histopathology (HP), which contains keratotic epithelium, basilar degeneration, a sawtooth appearance of the rete ridges as well as a dense subepithelial lymphocytic band with the presence of Civatte bodies (degenerating keratinocytes) [2]. In contrast to OLP, in oral lichenoid lesions (OLL), HP presents a slightly different picture consisting of mixed inflammatory infiltrate with a higher frequency of plasma cells, lymphocyte exocytosis extending further into the suprabasal layers and deeper into the lamina propria with the presence of perivascular inflammation. 

However, oral lesions in many patients do not present a highly specific image on standard light microscopy, thus it should be supplemented by immunological tests. 

Direct immunofluorescence (DIF) is a gold technique used to differentiate between OLP and other diseases manifested by oral erosions, such as autoimmune blistering diseases [3]. When examining LP cases, DIF reveals an irregular deposition of fibrinogen along the basement membrane zone (BMZ), with a lack of immunoglobulins [1]. It is worth noting that the presence of fibrinogen deposition is not exclusive to OLP, as other oral potentially malignant disorders (OPMDs) may exhibit a similar pattern [2]. Furthermore, in oral lichenoid drug reactions, there can also be the occurrence of fibrin deposits at the BMZ and the presence of IgM-positive cytoid bodies, resembling those found in OLP [3]. 

The role of DIF in the diagnostics of OLP has been described since the 1980s in different populations [3,4,5,6,7,8]. According to our best knowledge, there are no similar data for Polish patients, although the high variability in the percentage of positive DIF results among available publications (62–100%) may indicate the involvement of factors related to ethnic origin. 

Therefore, the purpose of this study is to assess DIF in relation to the histopathologic examination in Polish patients with oral findings consistent with OLP.

## 2. Materials and Methods

### 2.1. Patients

This retrospective study reports findings from routine clinical practice involving adult patients with oral cavity lesions recruited between 2020 and 2023 at the Department of Immunodermatology. These patients were referred by dentists closely cooperating with our department and other dermatologists for the diagnostics of chronic oral lesions using HP and DIF. None of the patients was treated systemically due to oral lesions before diagnostics.

Initially, 78 patients consecutively referred to the outpatient clinic who presented with oral changes suggestive of LP (erosions, white patches, gingival desquamation in different configurations) were included in the study. Those who lacked any test results or refused to take a biopsy were excluded from the final analysis. 

Patients provided consent for the routine diagnostic procedures. Due to standard diagnostic procedures, approval from the ethics committee was not required.

### 2.2. Histopathology

Lesions in the oral mucosa were sampled using a 0.4 cm punch with local anesthesia containing lidocaine. The obtained sections were then fixed in formalin and transported to the laboratory. Following ethanol dehydration, the tissues were embedded in a paraffin block, cut using a microtome, and the resulting several-micrometer-sized scrapings were placed on standard slides. These slides were subjected to eosin and hematoxylin staining, covered with a coverslip, and subsequently analyzed under a light microscope.

### 2.3. Direct Immunofluorescence

Normally appearing oral mucosa was taken using a 0.4 cm biopsy punch under local anesthesia with lignocaine. These sections were then frozen in OCT (Lab-Tec Division Miles tissue freezing medium, IN, USA) at −30 °C and stored at −80 °C. Slices of equal thickness (6 μm) were obtained using a cryostat (−30 °C) from Reichert-Jung and then transferred to primary slides. After drying at room temperature for 30 min, the slides were incubated for 30 min with antibodies against human IgG (Cappel), IgA (Cappel), IgM (Cappel), C3 complement component (Cappel), and fibrinogen (we used two antibodies—F1 (Cappel) and F2 (Dako) bound to FITC). Following a 3 × 5 min wash in PBS, the sections were sealed in PPD (10 mL PBS, 100 mg paraphenylenediamine, and distilled water to 100 mL, pH 8.0) and examined under a fluorescence microscope, Leica DM1000 LED fluo.

### 2.4. ELISA for IgG Antibodies Directed Against the NC16a Epitope of the BP180 Antigen

To determine the concentration of antibodies directed against the NC16a epitope of the BP180 antigen, the BP180 ELISA kit from Medical & Biological Laboratories CO., Ltd. (Nagoya, Japan) was utilized according to the manufacturer’s recommendations. Calibrator 1 (k-1, negative control), calibrator 2 (k-2, positive control), and sera from patients in the test (BP patients) and control groups were applied to microwell plates coated with recombinant NC16a antigen. Each serum was tested in duplicate, and after incubation and washing, horseradish peroxidase-labeled goat antibodies directed against human IgG were added. Subsequent steps involved additional washes, substrate addition, incubation, and termination of the reaction. Optical density at 450 nm (A450) was measured, and results were expressed as an index value (in units of U/mL) according to the manufacturer’s protocol.

### 2.5. Statistical Analysis 

Statistical analysis, performed using Statistica 13, included the presentation of qualitative variables as numbers and percentages. Chi-square (Fisher exact) tests were used to assess statistical significance, with a significance level set at 0.05. Diagnostic test quality indicators were calculated using the Diagnostic Test Evaluation Calculator (MedCalc Software v. 23.0.9) and expressed as percentages with 95% confidence intervals.

## 3. Results

Eventually, 66 patients participated in the study (17 [25.8%] males and 49 [74.2%] females). Among them, 51 (77.3%) had erosions, and 51 (77.3%) exhibited ulcerations within the oral cavity (in any specific location). Bilateral erosions on the buccal mucosa were observed in 36 (54.5.0%) patients, and bilateral white patches on the buccal mucosa were found in 39 (59.1%) patients. Clinical features are depicted in Table 1. 

In our examined group, 33 patients (50.0%) received a histopathological finding confirming the diagnosis of OLP (Group 1), and the remaining 33 patients (50%) did not fulfill the HP features of OLP (Group 2).

### 3.1. Group 1—Contains Patients in Whom HP Was Characteristic of LP

As Table 2 shows, in this group, DIF-IgG and DIF-IgA examinations were negative in all cases.

**Figure 1:**
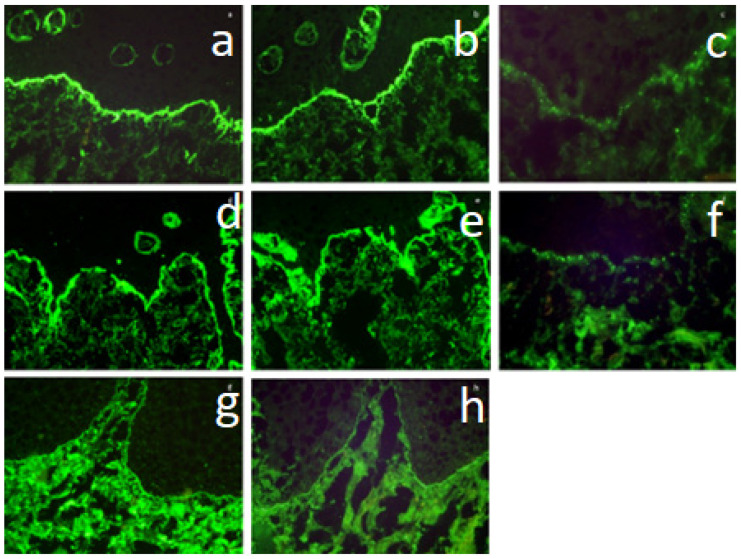
(**a**–**h**). Upper row—LP confirmed in histopathology: (**a**) strong deposition of F1 in a linear pattern along the BMZ (DIF, ×200), (**b**) strong deposition of F2 in a linear pattern along the BMZ (DIF, ×200), (**c**) deposition of complement C3 in a granular pattern along the BMZ (DIF, ×400). Middle row—LP-like lesions without LP histopathology: (**d**) strong deposition of F1 in a linear pattern along the BMZ (DIF, ×200), (**e**) strong deposition of F2 in a linear pattern along the BMZ (DIF, ×200), (**f**) deposition of complement C3 in a granular pattern along the BMZ (DIF, ×400). Lower row—MMP patient with LP-like lesions: (**g**) strong deposition of IgG in a linear pattern along the BMZ (DIF, ×200), (**h**) deposition of linear complement C3 along the BMZ (DIF, ×200). LP-lichen planus, DIF—direct immunofluorescence, F1-fibrinogen 1, F2—fibrinogen 2, BMZ—basement membrane zone, MMP—mucous membrane pemphigoid.

DIF-F1 examination in shaggy arrangement was positive in 14 patients (42.4%) (Figure 1a) and negative in 19 cases (57.6%).

DIF-F2 examination in shaggy arrangement was positive in 12 patients (36.4%) (Figure 1b) and negative in 21 cases (63.6%).

DIF-C3 examination in granular arrangement was positive in 6 patients (18.2%) (Figure 1c) and negative in 27 cases (81.8%).

DIF-IgM examination in linear arrangement was positive in one patient (3.0%) and negative in 32 cases (97.0%).

ELISA for BP180 was negative in all cases in this group (100%).

### 3.2. Group 2—Contains Patients in Whom HP Did Not Detect LP Features

In this group, the DIF-F1 examination was positive in 7 cases (21.2%, all shaggy patterns) (Figure 1d) and negative in 26 patients (78.8%).

DIF-F2 examination was positive in 6 cases (18.2%, all shaggy pattern) (Figure 1e) and negative in 27 patients (81.8%).

DIF-C3 examination was positive in 6 cases (18.2%, including granular arrangement in 4 cases [12.1%] and linear pattern in 2 cases [6.1%]) (Figure 1f) and negative in 27 (81.8%) patients.

DIF-IgG examination was positive in 2 cases (6.1%, linear arrangement in both) (Figure 1g) and negative in 31 (93.9%) patients. 

DIF-IgA was positive in one case (3.0%, linear arrangement) (Figure 1h) and negative in 32 (97.0%) patients. 

DIF-IgM examination was positive in one case (3.0%) (linear arrangement) and negative in 32 patients (97.0%). 

ELISA for BP180 was positive in 2 (6.1%) cases (both cases had a linear pattern in DIF-IgG) and negative in 31 (93.9%) patients.

Statistical analysis disclosed the DIF profile entirely typical for LP (C3+, F+) was present in 7 patients, among whom 4 (57.1%) had a positive HP result, and 3 individuals (42.9%) had a negative result. Overall, 14 patients with confirmed LP in HP had a positive result for F1, and 12 patients had a positive result for F2, constituting 42.4% and 36.4% of individuals with a positive HP result, respectively. No positive fibrinogen was observed in 26 (78.8%) patients with unspecific HP and 19 (57.6%) patients with HP characteristics of LP. Patients with positive fibrinogen included one patient (3.0%) with negative HP and two (6.1%) patients with positive HP. Both positive fibrinogens were present in 6 (18.2%) patients with negative HP and 12 (36.4%) patients with positive HP. All patients tested positive for F1 also had a positive F2 result simultaneously. DIF-C3 was positive in 6 (18.2%) patients (Table 2). Simultaneously, in 50% (6/12) of individuals with a positive DIF result for C3, it was confirmed by HP. Among 21 and 18 individuals with a positive DIF result for F1 or F2, respectively, the histopathological confirmation rate was 66.7% in both cases.

One patient had a profile characteristic of lupus (only IgM+) but with an HP result typical for LP. Two patients had positive C3 and IgG, with one of them also testing positive for IgA and the other for ELISA BP180, leading to the diagnosis of MMP. MMP was also diagnosed in a third patient based on linear fluorescence of C3.

The sensitivity, specificity, positive predictive value, negative predictive value, and overall diagnostic accuracy were highest for the presence of fibrinogen deposits, at 36% and 42%, 79% and 82%, 67% and 67%, 58% and 56%, and 61% and 59%, respectively (Table 3).

## 4. Discussion

The differentiation of entities resulting in oral white patches and ulcers primarily relies upon clinical presentation supported by HP; however, it is not always reliable. First, not all cases display the common clinical or histological symptoms linked to a particular condition. Second, HP examination can yield a false negative result when the early period of illness or the material is collected improperly. If a biopsy is taken from a central part of an ulcerated area, it usually shows an inflammatory infiltration without typical features of LP [9,10]. Therefore, it is suggested to take the biopsy from the edge of a classical plaque if OLP is suspected [9]. Moreover, lichenoid lesions usually caused by irritating agents may clinically mimic OLP. Eventually, patients with oral dysplastic lesions may present both clinical and histologic symptoms resembling OLP, and OLP can also show dysplasia [11,12]. Despite these limitations and objections, HP remains the first examination performed in patients with chronic oral lesions. Recognizing we compared its results with a full panel of immunoglobulins (IgG, IgA, IgM, C3, and fibrinogen) by DIF since recently it was postulated that a shaggy pattern of fibrinogen along BMZ in the absence of immunoglobulins and complement is the most characteristic picture in OLP [3,6]. In the current study, positive fibrinogen was observed in patients with HP picture conclusive for OLP, and it was twice as frequent as in patients who did not fulfill HP criteria of OLP with sensitivity below 50% in both groups. We also showed that among the antibodies we used, F1 was slightly more sensitive than F2, but both DIF-F1 and DIF-F2 tended to be associated with HP outcomes but without exceeding the threshold for statistical significance, whereas the other immunological tests showed no significant association with HP outcomes. A positive result in this test allowed confirmation of the disease with approximately 66% certainty, while a negative result definitively ruled it out with approximately 57% certainty. The overall diagnostic accuracy of this test in our experiment was around 60%. The predictive values of the other methods did not exceed 50%, with a notably greater usefulness for excluding the disease rather than confirming it. Our results are in contrast to results by Yamanaka et al. [13] and Korkitpoonnnpol et al. [11], who showed positive fibrinogen at the BMZ in most of their patients with OLP and lichenoid lesions as well. Differences in the sensitivity of fibrinogen in DIF between authors are due to different qualification conditions of patients in the studies. Our study was not intentionally focused on already diagnosed OLP but included consecutive patients with white patches in oral mucosa. However, we are a highly specialized referral center where patients with suspected OLP, identified based on their medical history and symptoms, are referred for advanced diagnostic evaluation. Nevertheless, in certain cases, histopathological examination does not confirm this diagnosis. This raises the question of whether this outcome reflects the absence of OLP or the limited specificity of the observed features within the collected tissue sample. Based on the characteristics in Table 1 and also low histopathological confirmation, it is highly probable that one part of the patients do not have OLP. The reason the results of this study contrasted with previous reports might be that Group 2 included non-specific oral lesions, while in previous reports, the control group was limited to the characterized conditions, like BP, MMP, and leukoplakia. Therefore, although the positive predictive value appeared low, DIF may still be helpful in diagnosing OLP. Nevertheless, our previous studies disclosed that DIF should not be the only test for diagnostics of OLP or lichenoid lesions since several cases might be missed [14].

An interesting finding in our study refers to a granular pattern of C3 along the BMZ in a few patients with HP typical for OLP; however, it was also present in patients with uncharacteristic HP pictures. A similar observation was made by Korkitpoonpol et al. [11], who also showed the presence of that pattern in oral epithelial dysplasia. Since the nature of that finding is unclear, diagnosticians may easily ignore it as non-specific. However, it was postulated that C3 plays a significant role in periodontitis [15], a chronic inflammatory disorder in the oral cavity, through activating inflammatory mediators leading to destructive periodontal tissue inflammation and bone loss in periodontitis. Since there is a correlation between periodontal inflammation and complement activation, C3 was postulated as a potential target of therapeutic intervention. Recent clinical trials showed that C3-targeted inhibition blocks gingival inflammation in patients with periodontal disease [16]. OLP may be the reaction to chronic inflammation in the oral cavity caused, for example, by periodontitis. This could explain the presence of granular C3 at the BMZ in OLP and lichenoid lesions. Further studies performed along with microbiological examination are needed for a better understanding of its real role.

Another valuable DIF result in our study is the early identification of a few patients with exclusive oral lesions as mucous membrane pemphigoid (MMP) based on a positive reaction with IgG, IgA, and C3 immunoglobulins. All three of these patients presented oral changes closely resembling OLP without skin involvement and HP without LP symptoms. One of these patients had both IgG and IgA at the BMZ, and the second had IgG, IgA, and C3 along the BMZ; the third presented exclusively C3 deposits at the BMZ, all characteristically of MMP. In two of three patients, the diagnosis of MMP was supported by positive ELISA for the NC16a antigen of BP180. Noteworthy, none of these three patients had positive fibrinogen along the BMZ. Prompt diagnosis of MMP enabled the initiation of proper treatment in them.

While the reliability of DIF in autoimmune bullous diseases (AIBD) is out of an argument, it has still been under debate in establishing a diagnosis of OLP [17,18,19]. It is widely accepted that DIF in AIBD is superior to histopathology, especially in clinically common cases. However, in cases of doubt, DIF can be helpful. On the other hand, some authors consider DIF unnecessary in the routine diagnosis of oral cavity lesions if clinical suspicion for an oral autoimmune bullous disorder is low [18,19,20]. 

In conclusion, our data indicated the complementarity of HP and immunological test results and the usability of using both methods in cases with oral lesions. In some of them, diagnostics should be supported by additional immunological assessments, like salt-split-skin testing, immunoblot/immunoprecipitation, and ELISAs, to establish a conclusive diagnosis and distinguish OLP from other blistering disorders, particularly MMP. However, these methods can be expensive, time-intensive, and not universally accessible.

## Figures and Tables

**Table 1:** Clinical characteristics of the patients.

Feature	Yes (%)	No (%)
Sex: male	17 (25.8)	49 (74.2)
Erosions	41 (62.1)	25 (37.9)
Bilateral erosions	36 (54.5)	30 (45.5)
Ulcerations	51 (77.3)	15 (22.7)
White patches	39 (59.1)	27 (40.9)
Histological confirmation of OLP	33 (50.0)	33 (50.0)

**Table 2:** The number and percentage of patients with DIF particular subclasses present or absent among those with positive or negative results of histopathologic examination.

Antibody Class	DIF Result	Type of Fluorescence	Histopathology		*p*-Value
			Positive N = 33	Negative N = 33	
DIF IgG	Positive (n = 2)	Linear (n = 2)	0 (0.0) 0 (0.0) 33 (100.0)	2 (6.1) 0 (0.0) 31 (93.9)	0.4923
		Granular (n = 0)			
	Negative (n = 64)				
DIF IgA	Positive (n = 1)	Linear (n = 1)	0 (0.0) 0 (0.0) 33 (100.0)	1 (3.0) 0 (0.0) 32 (97.0)	1
		Granular (n = 0)			
	Negative (n = 65)				
DIF IgM	Positive (n = 2)	Linear (n = 2)	1 (3.0) 0 (0) 32 (97.0)	1 (3.0) 0 (0) 32 (97.0)	1
		Granular (n = 0)			
	Negative (n = 64)				
DIF C3	Positive (n = 12)	Linear (n = 2)	0 (0.0) 6 (100.0) 27 (81.8)	2 (33.3) 4 (66.6) 27 (81.8)	1
		Granular (n = 10)			
	Negative (n = 54)				
DIF F1	Positive (n = 21)	Shaggy (n = 21)	14 (42.4) 0 (0) 19 (57.6)	7 (21.2) 0 (0) 26 (78.8)	0.118
		Granular (n = 0)			
	Negative (n = 45)				
DIF F2	Positive (n = 18)	Shaggy (n = 18)	12 (36.4) 0 (0) 21 (63.6)	6 (18.2) 0 (0) 27 (81.8)	0.0973
		Granular (n = 0)			
	Negative (n = 48)				
ELISA BP180	Positive (n = 2)	Linear (n = 2)	0 (0.0) 0 (0.0) 32 (100.0)	2 (6.1) 0 (0.0) 31 (93.9)	0.1571
		Granular (n = 0)			
	Negative (n = 63)				

**Table 3:** The reliability parameters (with 95% confidence intervals) of individual antibody subclasses used in DIF to establish the diagnosis of lichen planus in relation to the histopathologic examination results as the reference test.

Statistic	DIF IgG	DIF IgA	DIF IgM	DIF C3	DIF F1	DIF F2	DIF F1 + DIF F2	ELISA
Sensitivity	0.0 0.0–10.6	0.0 0.0–10.6	3.0 0.1–15.8	18.2 7.0–35.5	42.4 25.5–60.8	36.4 20.4–54.9	38.7 21.8–57.8	0.0 0.0–10.9
Specificity	93.9 79.7–99.3	97.0 84.2–99.9	97.0 84.2–99.9	81.8 64.5–93.0	78.8 61.1–91.0	81.8 64.5–93.0	81.2 63.6–92.8	93.9 79.8–99.5
Positive Predictive Value	0	0	50.0 6.1–93.9	50.0 26.4–73.6	66.7 48.1–81.2	66.7 46.0–82.4	66.7 46.2- 82.3	0
Negative Predictive Value	48.4 46.3–50.6	49.2 47.7–50.7	50.0 47.9–52.1	50.0 44.3–55.7	57.8 49.3–65.8	56.2 48.7–63.5	57.8 49.7–65.5	49.2 47.0–51.4
Accuracy	47.0 34.6–59.7	48.5 36.0–61.1	50.0 37.4–62.6	50.0 37.4–62.6	60.6 47.8–72.4	59.1 46.3–71.0	60.3 47.2–72.4	47.7 35.1–60.5

Sensitivity: The probability that a test result will be positive when the disease is present (true positive rate). Specificity: The probability that a test result will be negative when the disease is not present (true negative rate). Positive predictive value: The probability that the disease is present when the test is positive. Negative predictive value: The probability that the disease is not present when the test is negative. Accuracy: The overall probability that a patient is correctly classified.

## Data Availability

Source data are available upon request from the corresponding author.
